# *Thermus thermophilus* as a Source of Thermostable Lipolytic Enzymes

**DOI:** 10.3390/microorganisms3040792

**Published:** 2015-11-04

**Authors:** Olalla López-López, María-Esperanza Cerdán, María-Isabel González-Siso

**Affiliations:** Grupo EXPRELA, Centro de Investigacións Científicas Avanzadas (CICA), Departamento de Bioloxía Celular e Molecular, Facultade de Ciencias, Universidade da Coruña, Campus de A Coruña, 15071 A Coruña, Spain; E-Mails: olopez@udc.es (O.L.-L.); esper.cerdan@udc.es (M.-E.C.)

**Keywords:** *Thermus thermophilus*, lipase, esterase, lipolytic, thermophilic

## Abstract

Lipolytic enzymes, esterases (EC 3.1.1.1) and lipases (EC 3.1.1.3), catalyze the hydrolysis of ester bonds between alcohols and carboxylic acids, and its formation in organic media. At present, they represent about 20% of commercialized enzymes for industrial use. Lipolytic enzymes from thermophilic microorganisms are preferred for industrial use to their mesophilic counterparts, mainly due to higher thermostability and resistance to several denaturing agents. However, the production at an industrial scale from the native organisms is technically complicated and expensive. The thermophilic bacterium *Thermus thermophilus* (*T. thermophilus*) has high levels of lipolytic activity, and its whole genome has been sequenced. One esterase from the *T. thermophilus* strain HB27 has been widely characterized, both in its native form and in recombinant forms, being expressed in mesophilic microorganisms. Other putative lipases/esterases annotated in the *T. thermophilus* genome have been explored and will also be reviewed in this paper.

## 1. Lipolytic Enzymes

The term “lipolytic enzymes” include esterases (EC 3.1.1.1) and lipases (EC 3.1.1.3). They catalyze the breaking of ester bonds between alcohols and carboxylic acids in aqueous media. In organic solvents or low water content media they can catalyze the formation of ester bonds by the reactions of esterification or transesterification. Lipolytic enzymes are chemoselective, regioselective and enantioselective [1].

Esterases and lipases are distinguished by substrate specificity. The substrates of esterases are water-soluble esters with lateral chains shorter than 10 C, while lipases prefer water-insoluble substrates with lateral chains longer than 10 C. Moreover, lipases, but not esterases, show interfacial activation, which is due to the presence of a hydrophobic lid in most lipases that covers the active center in the closed inactive conformation of the enzyme. In the open conformation the active center is accessible for the substrate and the enzyme is active. The transition between closed to open forms occurs in the presence of a lipidic interphase or a substrate emulsion that opens the lid, as a consequence of which the enzyme is activated. Esterases follow classical Michaelis–Menten kinetic behavior [2].

Lipolytic enzymes are widely distributed in nature, being present in animals and plants, where their physiological function is the hydrolysis of triglycerides, as well as in fungi and bacteria. Microbial enzymes are highly versatile, able to catalyze a variety of reactions, do not need cofactors and are stable and active in organic solvents [1,2]. These characteristics, together with the short generation time of producing microorganisms and ability of genetic manipulation, scale up and protein purification, explain why microbial enzymes are the highly valued in the industrial field [3].

**Figure 1:**
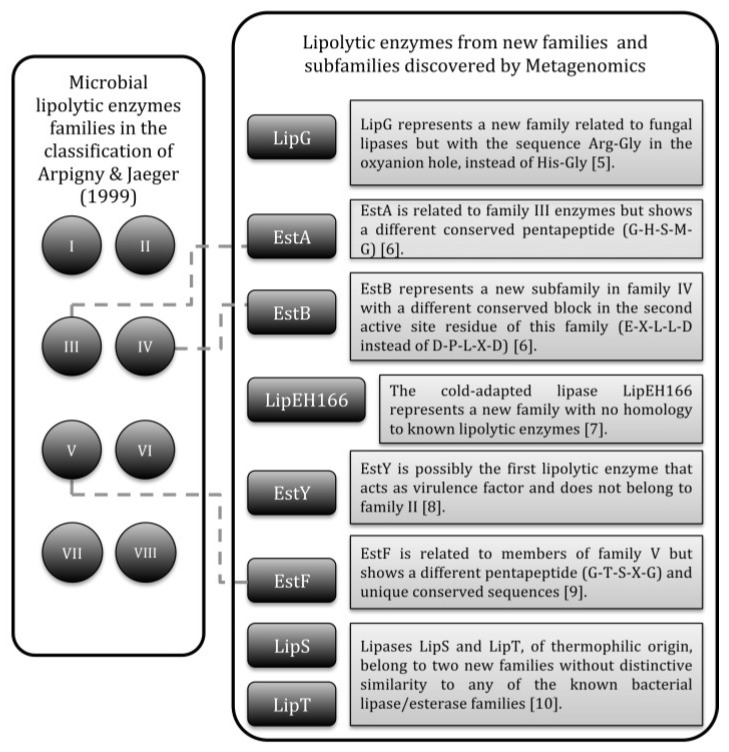
Bacterial lipolytic enzymes that represent new families or subfamilies added to the classification of Arpingy and Jaeger and discovered by metagenomics.

The classification of bacterial lipolytic enzymes by Arpingy and Jaeger [4] continues to be mostly used nowadays. The enzymes were initially clustered in eight families with subfamilies in function of conserved motifs and biological properties. Over time, new families and subfamilies were added to the original classification, some of them coming from metagenomics studies (Figure 1) [5,6,7,8,9,10], *i.e.*, studies of genomes directly extracted from environmental microbial populations, including non-culturable organisms [11,12].

### 1.1. Structure and Mechanism

**Figure 2:**
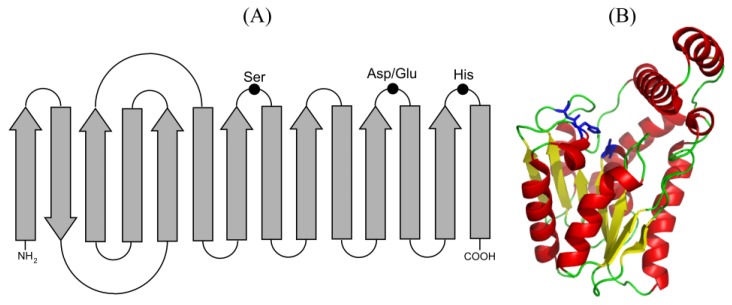
(**A**) Representation of the secondary structure of the α/β hydrolase fold showing the residues involved in catalysis. α helices and β strands are shown as arrows and boxes, respectively, black dots represent the residues of the catalytic triad. (**B**) Crystallographic structure of the carboxylesterase Est30 from *Geobacillus stearothermophilus* (PDB ID:1TQH) showing a modified α/β hydrolase core with a seven-stranded β sheet and a cap domain comprising three alpha helices. The ribbon diagram is colored yellow for β strands, red for α helices and green for loops. Residues of catalytic triad are shown as sticks in blue.

Lipolytic enzymes show low homology in their primary sequence but share structural motifs and catalytic mechanism. They present the α/β hydrolase fold that consists of eight parallel β-sheets, except the second one that is anti-parallel, connected by six α-helixes that contain the active center [13]. The active center is formed by the catalytic triad Ser-Asp/Glu-His, with the Ser generally embedded in the consensus sequence G-X-S-X-G. This nucleophilic Ser immediately follows the 5β sheet, the acid residue (Asp/Glu) is located in a turn after the 7β sheet and the His residue is in the loop after the 7β sheet. Another two amino acids, one located next to the catalytic Ser and the other at the end of the 7β sheet, are part of the oxyanion hole and contribute to the stabilization of the intermediates of the enzymatic reaction (Figure 2). Moreover, following the β-sheets placed at the C-terminal half of the enzyme, the regions responsible for substrate accommodation and substrate specificity have been identified. There are lipolytic enzymes with folding variants, with differences of the consensus sequence surrounding the catalytic Ser, or with high homology to non-lipolytic enzymes [1,13].

The catalytic mechanism of lipolytic enzymes starts with the nucleophilic attack of the catalytic Ser hydroxyl to the carbonyl of the ester bond. A tetrahedral intermediate is thus formed, which is stabilized by the macrodipole of the αC helix, by the catalytic residues His and Asp/Glu and by the NH groups of the peptide chain that is part of the oxyanion hole. Then, the alcohol is cleaved and the acyl-enzyme complex is formed. The next step is de-acylation: The nucleophilic attack either of a water molecule (hydrolysis reaction) or of an alcohol or ester (esterification or transesterification reactions) forms a new tetrahedral intermediate that will be cleaved into the product, acid or ester, and the free enzyme [1,2].

### 1.2. Applications

The lipolytic enzymes represent about 20% of the world enzyme market that is estimated to reach 7.6 billion dollars in 2015 [14], which clearly indicates the economic importance of lipases and esterases. These enzymes are able to efficiently transform a wide range of natural and non-natural substrates in aqueous and organic media [15]. In addition to organic solvents, other reaction media more respectful of the environment have been recently developed, such us “ionic liquids” [16,17] and “deep eutectic solvents” [18].

**Figure 3:**
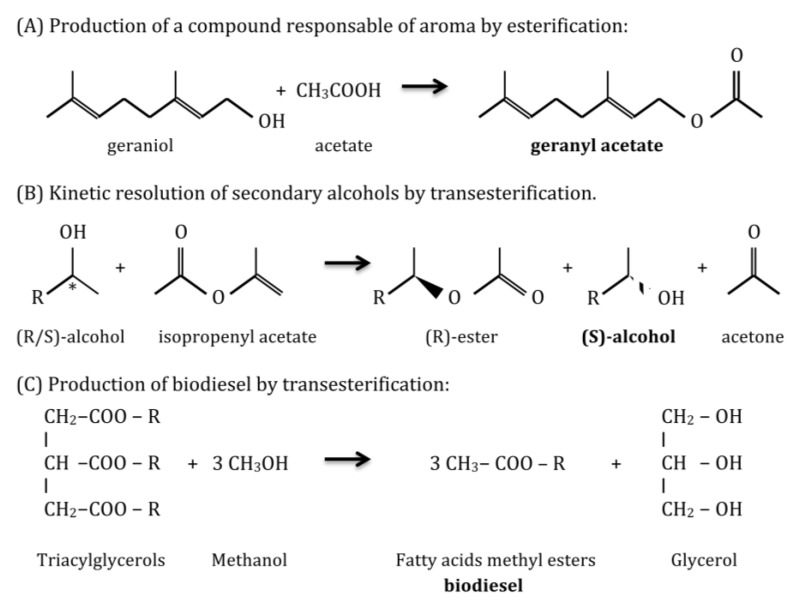
Examples of applications of lipase/esterase catalyzed reactions: (**A**) production of geranyl acetate ; (**B**) enantiopure secondary alcohols and (**C**) biodiesel.

Reactions catalyzed by lipolytic enzymes are necessary in a variety of industrial branches (Figure 3). Thus, lipases and esterases are used: in the food industry to produce flavoring esters, sugar esters that act as emulsifiers, and to modify the fatty acid composition of triglycerides, in order to reduce the risk of cardiovascular disease, by introducing omega-3 or removing saturated fatty acids, exploiting their chemoselectivity [3,19]; in the pharmaceutical industry to resolve racemic mixtures for drug synthesis, due to their high enantioselectivity, to produce biologically active enantiomers [19,20]; in the cosmetic and perfumery industry to produce by esterification mono- and diacylglycerols that are employed as surfactants and aroma responsible compounds [21]; in the paper industry to remove resins from paper pulp [20]; in detergents industry as additives to remove grease stains in combination with proteases and cellulases [20]; in clinical diagnosis lipases can be used as biomarkers to detect pancreatitis or tuberculosis infection [3,20]; in analytical assays for specific DNA sequences as reporter genes coupled with chromogenic substrates [3]; in biodiesel production, as an alternative to alkaline chemical catalysis that is harmful to the environment, by transesterification of oils in methanol or a different alcohol to produce alkyl esters of fatty acids [19]; in the synthesis of biopolymers from structurally complex monomers, due to their high regio-, chemo-, and stereoselectivity [3]; and finally in waste treatment and bioremediation of oil spills [20].

## 2. Lipolytic Enzymes from Thermophilic Microorganisms

The cost of these enzymes is a key factor in the economy of every industrial process and these costs are highly influenced by the stability of the enzymes, *i.e.*, the duration of their operational activity levels [22]. Industrial bioprocessing at high temperatures shows advantages such as a reduced risk of contamination by mesophiles or an increased reaction rate due to a decrease of viscosity and an increase of the diffusion coefficient and the solubility of the substrate [23,24]. Therefore, highly robust enzymes are required by the industry, and they are being sought largely among thermophilic microorganisms.

Thermophilic microorganisms, and the enzymes they produce, are adapted to the life at high temperatures, their optimal growth temperature being 45–80 °C [25]; those with optimal growth temperatures higher than 80 °C are called hyperthermophiles. Other extremophilic conditions such as high salinity, high pressure, acid or basic pH, usually accompany high temperature in the same environment.

To be able to survive at these high temperatures, thermophiles have evolved modifications such as, for example, cellular membranes enriched with saturated fatty acids that maintain the cells more rigid, or DNA reverse gyrases that produce supercoiled DNAs with melting points in the range of the temperature necessary for the microorganism’s growth [23].

Regarding protein structure and composition, several individual factors, which are different for each protein, but not a common strategy, have been identified as being responsible for improving stability at high temperatures. Some of these factors, amongst others, are grouped charges, additional hydrogen bridges, packing optimization and reduction of number and size of internal cavities, increase in the number of charged amino acids at the expense of uncharged polar residues, concealment of hydrophobic surfaces and the more hydrophobic nucleus, increase of hidden area by oligomerization, avoidance of the use of thermo-unstable residues, increase of secondary structures (helix), formation of salt bridges mainly at the surface, and increase of Proline content [26,27,28,29]. Thermophilic lipolytic enzymes present similar adaptations as other thermophilic proteins. Additionally, an increase in inverse gamma turns has been detected in the N- and C-terminus of the α-helixes and β-sheets [30].

All these adaptations lead to an increase of conformational rigidity and compactness of the protein structure at mesophilic temperatures and at the same time to the maintenance of a suitable flexibility for catalysis at high temperatures [31].

There are applications that benefit from thermostable enzymes but cannot be performed at high temperatures, such as some reactions in fine chemistry due to substrate instability, or the use of detergents to wash at cold temperatures. Hence new lipolytic enzymes that are thermostable but active at low temperatures are being sought for the industry. The enzymes for detergents also have to be resistant to an alkaline pH [20]. However, most industrial processes where lipases are used are performed at temperatures higher than 45 °C [23], for example to modify substrates that are solid or insoluble at ambient temperature [32], or to enzymatically produce biodiesel [33]. Thermophilic lipolytic enzymes with specific characteristics for each process are demanded by the industry.

The thermophilic microorganisms cited in the literature to produce lipases or esterases include species of archaea such as *Pyrococcus furiosus* and *Thermotoga* sp., bacteria such as *Bacillus thermocatenulatus* and *B. stearothermophilus*, and fungi such as *Geotrichum* sp. and *Thermomyces lanuginosus* (*T. lanuginosus*), amongst others [24].

### Expression and Production

The production of enzymes at an industrial scale from thermophilic microorganisms presents several drawbacks such as low yields of the enzymes, slow growth rates of the microorganisms, specific nutrient requirements, the need to heat the fermenters to maintain the optimal growth temperature, culture medium evaporation, corrosion of equipment, and decrease of solubility of gases [34]. Moreover, lipases are usually produced at late growth phases [23].

The possibility to clone the genes that encode thermophilic lipolytic enzymes under the control of strong promoters in order to overexpress the enzymes in mesophilic organisms, which are of faster growth and easier to culture than the native thermophilic ones, is the most efficient alternative to produce these enzymes at an industrial scale. In addition, the system for heterologous expression can lead to the secretion of the enzyme into the culture medium, thus facilitating its downstream processing and purification.

The bacterium *Escherichia coli* and the yeast *Saccharomyces cerevisiae* (*S. cerevisiae*) are the most frequently employed microorganisms for producing recombinant proteins at a high scale [34]. There are genetically modified strains of *S. cerevisiae* that have been improved for the secretion of recombinant proteins thus facilitating downstream processing. Other species of yeasts, fungi and bacteria have shown suitable performances for industrial scale production [25]. However, the main drawback in general is that a fraction of enzymes of thermophilic origin is not properly folded when synthesized at mesophilic temperatures, and thus thermostability and activity can be affected [35]. For these cases, *Thermus* strains have been developed for being used as tools for overexpression of thermophilic proteins, although these strains have not proven useful for industrial scale production hitherto [36].

Nowadays, commercially available thermostable lipolytic enzymes are usually obtained from fungal or bacterial microorganisms that produce them extracellularly in the culture medium. One representative example is the product Lipolase 100L, commercialized by Novozymes, that consists of the *T. lanuginosus* enzyme expressed in *Aspergillus niger* [14]. Lipolase^®^ was the first commercialized recombinant lipase, since 1994 [22].

Both solid-state fermentation (SSF) and submerged fermentation (SmF) processes are being used at present for production of thermophilic lipolytic enzymes. Submerged fermentation is generally chosen because of greater control on growth conditions [25]; however, during last years, increased attention is being paid to extracellular lipases/esterases production using industrial wastes and agricultural residues by SSF [14,22]. The SSF process involves the cultivation of microorganisms on moist solid supports [22]. SSF compared to SmF for thermophilic enzyme production requires significantly lower capital investment, is a more simple technique with reduced energy requirements, low wastewater output, improved product recovery and higher volumetric productivity; a reduction in catabolite repression has also been reported to be another advantage of SSF process [14]. Filamentous fungi are the best-adapted species for SSF since SSF resembles their natural habitat [22]. One drawback of SSF is that there are still few bioreactor types suitable for this process at large scale, mainly due to insufficient mass and heat transfer [22,37].

Low-cost/residual materials used as culture media for lipolytic enzymes production include by-products of oil extraction from seeds (oil cakes), by-products of lignocellulosic origin like soy husk or sugarcane bagasse, industrial effluents produced from edible oil refinery, slaughterhouses and dairy products industry, like olive mill waste water and palm oil mill effluent, among others [22].

The methods used for purification of lipases/esterases are non-specific: extraction, precipitation, hydrophobic interaction and other types of chromatography; the required quality of the enzyme purification depends on its application and the highest is needed when used in synthesis reactions for the pharmaceutical industry [24].

## 3. *Thermus thermophilus* (*T. thermophilus*)

*Thermus* genus belongs to the Domain *Bacteria*, Phylum *Deinococcus-Thermus*; it is one of the most abundant genera among thermophilic bacteria. More than one hundred strains have been isolated from diverse ecosystems with extremely high temperatures [36,38]. *Thermus* bacteria are Gram-negative and grow aerobically at a high rate in complex media with optimal growth temperatures between 62 and 75 °C [36].

The species *T. thermophilus* is of special interest. It is a bacillus that produces yellow pigments and is devoid of motility. It was firstly isolated from a thermal spring in Japan [39] and preferentially lives in thermal water, from 47 to 85 °C, with a neutral or alkaline pH [40]. Several strains are also halotolerant since they have been isolated from marine locations [41].

The genomes of four *T. thermophilus* strains have been sequenced and are publicly available [42]. They show a high GC content (69%) and a high coding density (95%) [36].

Several thermostable DNA polymerases from *Thermus* sp., in addition to the pioneering Taq DNA polymerase, are being commercialized for use in the PCR technique [43]. *T. thermophilus* produces a series of enzymes of biotechnological interest, including glucose isomerase, xylose isomerase, proteases, beta-glucosidase, L-asparaginase, phosphatases (at least four), pyrophosphatase and several DNA and RNA processing enzymes [40]. More recently other promising enzymes from *T. thermophilus* have been reported such as NADH-oxidases [44], mannose-6-P-isomerase [45,46] and superoxide dismutases [47], amongst others.

*T. thermophilus* is also a source of new thermostable lipolytic enzymes with potential industrial uses as described below.

## 4. Lipolytic Enzymes from *Thermus thermophilus* HB27

The presence of lipolytic enzymes in *Thermus* sp. has been known for long time [48], but the first quantitative data of production were not reported until 2004 by Domínguez *et al.* [49]. These authors identified the strains *T. aquaticus* YT-1 and *T. thermophilus* HB27 to be the two best producers. Although lipolytic activity of thermophiles is usually mostly intracellular, these strains showed remarkable levels of extracellular activity. The lipolytic activity present in protein extracts of these strains resulted in a high thermostability (75%–100% activity remaining after 30 min at 80 °C) showing substrate preference for *p*-nitro-phenyl-esters of medium length fatty acids chains. By zymography analysis, two lipolytic enzymes of 34 and 62 kDa were detected both in intra- and extra-cellular fractions, with the 34 kDa enzyme being more abundant [50].

The 34 and 62 kDa lipolytic enzymes were partially purified from *T. thermophilus* HB27 and the enzymatic extract was characterized, showing optimal conditions for lipolytic activity at an alkaline pH and 80 °C [50]. Later, the 34 kDa enzyme was purified and characterized, showing a substrate preference for medium length chain esters, typical of esterases, with an optimal activity at pH 8.5 and 80 °C, and a half-life of 135 min at 85 °C; a 3D model obtained by threading was also published [51].

To improve growth and secretion of lipolytic enzymes by *T. thermophilus* HB27, the medium composition and culture conditions were optimized, both in Erlenmeyer flasks and fermenter cultures [52,53,54,55]. A positive effect on lipolytic enzyme production was observed when disaccharides were used as carbon source and when olive or sunflower oils were added to the culture medium [52,54]. The increase in lipolytic enzyme production obtained by replacing distilled water with natural thermal spring water in the culture medium is worth noting [55,56]. The secretion of the lipolytic enzymes was favored by the addition of surfactants at the late exponential growth phase [57]. However, in spite of all these advances, the growth and yield continued to be too low for the implementation of an industrial scale production process, and the lipolytic activity appeared to be largely retained within the biomass. The recombinant over-expression of the *T. thermophilus* HB27 lipolytic enzymes was accomplished, from mesophilic hosts with suitable properties for production of the enzymes, as described below.

### 4.1. Heterologous Expression of the 34 kDa T. thermophilus Esterase

Among the predicted open reading frames (ORFs) in the *T. thermophilus* HB27 genome database, the locus TT_C0904, annotated to encode a putative esterase of 329 amino acids and 36.0 kDa with the reference WP_011173331, showed the most similar characteristics to the previously purified 34 kDa esterase. The identity of WP_011173331 as the 34 kDa esterase was later corroborated by Matrix assisted laser desorption ionization—time of flight—mass spectrometry (MALDI-TOF-MS) of the *T. thermophilus* HB27 purified protein [51]. The esterase WP_011173331 was classified as a new enzyme family LipT, extending the existing classification according to Arpigny and Jaeger [10].

Our research group undertook the task to construct a mesophilic recombinant microbial strain with well-established culture techniques that are able to produce high levels of the protein in its active form that is also easy to purify. We assayed four different expression hosts, *E. coli* and three yeasts, with the full-length protein or two N-terminal deletions, as summarized in Table 1 where a comparison of some properties of the recombinant and native proteins is shown. The yeast species chosen for expression (*S. cerevisiae*, *Kluyveromyces marxianus* and *K. lactis*) have the GRAS (generally regarded as safe) status. *E. coli* and *S. cerevisiae* strains were selected as hosts because they are well known and have good performance plasmidic expression systems available; *K. lactis* was selected as host because an integrative, and therefore theoretically more stable, expression system is available; moreover producing secreted proteins and with lower glycosylation than those obtained in *S. cerevisiae*. In the case of the host *K. marxianus*, the 34 kDa *T. thermophilus* esterase was used as a model protein to develop an expression system and study the suitability of this yeast for heterologous thermophilic protein secretion in comparison with the other two yeast species [58,59,60,61].

All the 34 kDa *T. thermophilus* esterases forms in Table 1, native and recombinant, showed a preference for medium chain length substrates (C10) and an alkaline optimum pH, with the exception of E34Tt-His_6_ whose optimum pH was 6.3. The most alkaliphilic esterase form (optimum pH 9.6) was expressed in the *K. marxianus* system and localized in the cell wall fraction [58]. The recombinant proteins from the yeast strains were all glycosylated as expected for eukaryotic expression systems. The drop in the optimum temperature for activity shown by the recombinant 34 kDa *T. thermophilus* esterase forms compared to the native protein, ranging from 22 (E34Tt-His_6_) to 40 °C (ScEST3-O and KLEST-5A) depending on the expression system, is remarkable. The studies with ScEST3-O discarded the glycosylation and the tag added for affinity purification as causes of the decreased optimum temperature [59] that was attributed to improper folding [60]. The different thermostability values (measured as half-life at 85 °C) found for the different proteins in Table 1 are also remarkable. Thermostability of the full-length proteins, both native and recombinant, expressed by *E. coli*, increased in the presence of the detergent CHAPS (3-((3-cholamidopropyl) dimethylammonio)-1-propanesulfonate), while on the contrary, thermostability of the recombinant isoforms ΔN16 and ΔN26 decreased in the presence of CHAPS. CHAPS is generally used to solubilize hydrophobic membrane proteins when it is important to maintain protein activity. The N-terminal segment of the protein is hydrophobic, with the first 16 N-terminal residues being predicted to act as a secretion signal peptide (in the yeasts expression systems it was replaced by the secretion signal peptide included in the vector) and the region up to the 26th residue containing a predicted transmembrane helix. This hydrophobic N-terminal region was inferred to influence the degree of oligomerization and aggregation of the proteins [60].

The recombinant proteins ScEST3-O and KLEST-3S showed the highest levels of thermostability and also activity in the culture medium (extracellular production), and both features greatly improved in comparison to the native strain. Thus, total activity (extra- and intra-cellular) was about 30 and 50 times higher, respectively, and half-life at 85 °C in the absence of CHAPS was 14 and 12 times higher, respectively. The above exposed characteristics of the proteins ScEST3-O and KLEST-3S, together with the facilities for culturing the yeasts, made these strains excellent expression systems for high scale production of the 34 kDa *T. thermophilus* esterase in recombinant form.

**Table 1:** Heterologous expression systems of the 34 kDa *Thermus Thermophilus* (*T. thermophilus*) esterase and characteristics of the recombinant proteins in comparison with the native protein.

Name	Microorganism	Expression Vector	N-terminus	Optimum pH	Optimum Temperature	Half-life at 85 °C	Extracellular Production ^(1)^	Reference
E34Tt (Native)	*Thermus thermophilus* HB27	-	Full-length	8.5	80 °C	19 min; 135 min with 1% CHAPS	42% 120 U/L	[56]
ScEST3-O	*Saccharomyces cerevisiae* BJ3505	YEpFLAG-1	ΔN16	8.5	40 °C	260 min	<20% 1700 U/L	[59]
Km-EP	*Kluyveromyces marxianus* SLC33	pSPGK1	ΔN16	8.5–9.6	50 °C	102–108 min	45% 263 U/L	[58]
Km-IN	*Kluyveromyces marxianus* SLC33	pNADFL11/INU1	ΔN16	8.5–9.6	50 °C	102–108 min	50% 328 U/L	[58]
Kl-EP	*Kluyveromyces lactis* PM5-3C	pSPGK1	ΔN16	8.5	45 °C	84–180 min	12% 345 U/L	[58]
Kl-IN	*Kluyveromyces lactis* PM5-3C	pNADFL11/INU1	ΔN16	8.5	45 °C	84–180 min	13% 287 U/L	[58]
KLEST-3S	*Kluyveromyces lactis* NRRL-Y1140	pKLAC1	ΔN16	7.5	47.5 °C	230 min; 5 min with 1% CHAPS	98% 15000 U/L	[60]
KLEST-5A	*Kluyveromyces lactis* NRRL-Y1140	pKLAC1	ΔN26	8.5	40 °C	12 min; 2 min with 1% CHAPS	98% 600 U/L	[60]
E34Tt-His_6_^(2)^	*Escherichia coli* BL21(DE3)	pET-21d(+)	Full-length	6.3	58.2 °C	107.9 min with 1% CHAPS	<6% <5 U/L	[61]

**^(^**^1)^ Extracellular = into culture medium; Percentages represent extracellular *versus* total (intra- plus extra-cellular) activity. Lipolytic activity (U/L) was determined in all cases using *p*-nitrophenyl laurate as substrate and following the same method, the one described in [50]; **^(^**^2**)**^ In this case intracellular enzyme was used for characterization since only traces of activity were present in the culture medium.

### 4.2. Heterologous Expression of the T. thermophilus Putative Lipase WP_011173992

Among the loci predicted in the screening of the *T. thermophilus* HB27 database using the keywords lipase and esterase, the locus TT_C1623 attracted our attention because it was the only locus annotated to encode a putative lipase and because it is a small protein (18 kDa). Bioinformatic analysis of the sequence, using tools available in the ExPASy Proteomics Server (http://www.expasy.ch), revealed the absence of glycosylation sites (NetNGlyc 1.0 Server) and secretion signal peptide (Signal P 3.0 Server) as well as the hydrophobic character of the protein (ProtParam). We expressed this protein using the *S.cerevisiae*/YEpFLAG-1 system [62]. The recombinant protein could be detected by Western blot in the extra- and intra-cellular fractions, but the resulting lipolytic activity was very low, in fact scarcely detectable (less than 10 U/L measured with the same method cited in Table 1), even after the optimization of the conditions for measurement (effect on activity was assayed for pH from 4.5 to 8.5, temperature from 40 to 85 °C and substrate chain length from C6 to C12 *p*-nitrophenyl esters). In spite of being annotated as a putative lipase in the *T. thermophilus* HB27 genome database (reference AE017221), a BLAST search showed conserved motifs typical of phosphatases of the haloacid dehydrogenase (HAD) superfamily. Hence, we measured activity with *p*-nitrophenyl phosphate as substrate and we found significant three-fold higher activity values in the culture medium of the recombinant *S. cerevisiae* than in the medium of the untransformed yeast used as control. Medium of control strains (either untransformed or transformed with the void plasmid) usually give background values of activity that may be due to partial substrate autohydrolysis at high temperature or to some degree of activity of native mesophilic enzymes. The substrate specificity and optimum conditions for activity of this enzyme require further research. Since the recent update of the *T. thermophilus HB27* sequence database (reference NC005835), WP_011173992 is annotated as “haloacid dehalogenase” instead of lipase. However, other authors immobilized this WP_011173992 protein, cloned from *T. thermophilus* WL and expressed from *E. coli*, on supermagnetic nanoparticles and proposed the immobilized enzyme for practical application as a lipase [63]. Advantages of the immobilized lipase in comparison to the free one were the following: it could be separated from the reaction medium easily in a magnetic field, it exhibited better resistance to temperature, pH, metal ions, enzyme inhibitors and detergents, and it showed good reusability over 10 consecutive cycles, retaining 79.5% of its initial activity.

### 4.3. Functional Screening of T. thermophilus Lipolytic Enzymes Expressed in E. coli

**Figure 4:**
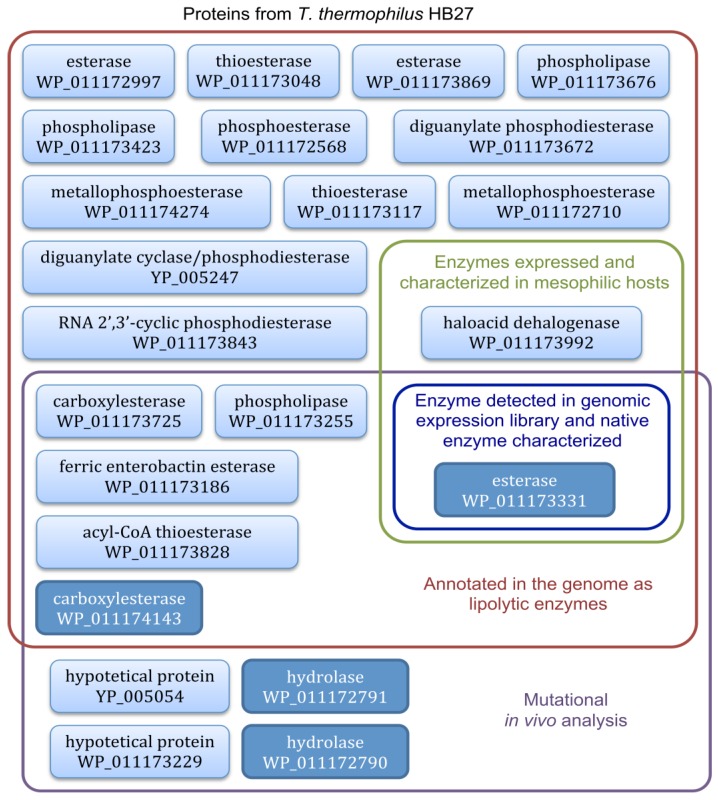
Scheme of predicted proteins from *T. thermophilus* HB27 (genome reference NC005835) studied hitherto to analyze its lipolytic activity and type of assay used. Boxes in dark blue correspond to the proteins identified to be mainly responsible for the lipolytic activity in *T. thermophilus* HB27. Boxes using former nomenclature (YP 005247 and YP 005054, genome reference AE017221) correspond to records that have been removed after the recent genome annotations update.

To ascertain whether other *T. thermophilus* HB27 loci encoding putative lipolytic enzymes could be expressed in active forms in mesophilic host, we constructed a genomic library of about 1000 clones in the vector pCC1FOS and revealed the positive lipolytic clones in solid medium with tributyrin. Sequencing of the two positive clones isolated from the library showed that they both contained the locus TT_C0904 encoding the previously characterized 34 kDa esterase that is responsible for the activity [62]. There is another example with a library constructed in plasmids from the *Thermus scotoductus SA-01* genome, which revealed a new enzyme named Est1 and belonging to a new family of lipolytic enzymes, which shares 76% sequence identity with the putative hydrolase of *T. thermophilus* HB27 WP_011172791 [64]. However, we did not isolate this putative hydrolase from the functional screening of our library. A recent genetic analysis by mutation of the genes encoding putative lipolytic enzymes in *T. thermophilus* HB27 showed that 75% of the activity was dependent on four loci [65]; we only detected one of them (Figure 4) which probably means that the others are not compatible with expression in *E. coli*. It has been reported that about 40% of thermophilic proteins are not expressed in active form from mesophilic hosts [66]. The *T. thermophilus* esterase-diminished strain [65] was unable to grow on minimal medium with tributyrin as sole carbon source and was used as a host to screen for metagenomic DNA fragments that complemented growth on tributyrin; a greater number of active esterase clones were scored in the thermophilic bacterium than in the mesophilic *E. coli* [67].

## 5. Conclusions

In the sequence of the genome of the thermophilic bacteria *T. thermophilus* HB27, multiple loci are predicted to encode putative lipolytic enzymes (Figure 4). One of them (TT_C0904 encoding the protein WP_011173331, characterized as an esterase) has been widely studied, it has been over-expressed and purified in a recombinant active form in the extracellular medium of mesophilic and industrially relevant hosts, thus greatly facilitating the production and downstream processing of the enzyme in comparison with the native producer. Among the different expression systems developed, two of them stand out, ScEST3-O and KLEST-3S, since they show the highest thermostability of the recombinant proteins and the highest levels of lipolytic activity secreted into the culture medium, both improving the native system.

The function of many other loci predicted to encode putative lipolytic enzymes has not been analyzed yet (Figure 4), and a mutant strain in several of them still shows lipolytic activity, which is a good indication that there are further till-date unknown lipolytic enzymes present in *T. thermophilus* HB27.
